# Bioactive fibrous scaffolds with programmable release of polypeptides regulate inflammation and extracellular matrix remodeling

**DOI:** 10.1093/rb/rbad010

**Published:** 2023-02-20

**Authors:** Zehong Xiang, Xinghua Guan, Zhifang Ma, Qiang Shi, Mikhail Panteleev, Fazly I Ataullakhanov

**Affiliations:** State Key Laboratory of Polymer Physics and Chemistry, Changchun Institute of Applied Chemistry, Chinese Academy of Sciences, Changchun, Jilin 130022, China; University of Science and Technology of China, Hefei, Anhui 230026, China; State Key Laboratory of Polymer Physics and Chemistry, Changchun Institute of Applied Chemistry, Chinese Academy of Sciences, Changchun, Jilin 130022, China; University of Science and Technology of China, Hefei, Anhui 230026, China; State Key Laboratory of Polymer Physics and Chemistry, Changchun Institute of Applied Chemistry, Chinese Academy of Sciences, Changchun, Jilin 130022, China; State Key Laboratory of Polymer Physics and Chemistry, Changchun Institute of Applied Chemistry, Chinese Academy of Sciences, Changchun, Jilin 130022, China; University of Science and Technology of China, Hefei, Anhui 230026, China; Key Laboratory of Polymeric Materials Design and Synthesis for Biomedical Function, Soochow University, Suzhou 215123, China; Dmitry Rogachev National Research Center of Pediatric Hematology, Oncology and Immunology, Moscow 117198, Russia; Faculty of Physics, Lomonosov Moscow State University, Moscow 119991, Russia; Dmitry Rogachev National Research Center of Pediatric Hematology, Oncology and Immunology, Moscow 117198, Russia; Faculty of Physics, Lomonosov Moscow State University, Moscow 119991, Russia

**Keywords:** bioactive scaffolds, inflammation control, extracellular matrix remodeling, polypeptide release, electrospinning

## Abstract

Inflammation manipulation and extracellular matrix (ECM) remodeling for healthy tissue regeneration are critical requirements for tissue engineering scaffolds. To this end, the bioactive polycaprolactone (PCL)-based scaffolds are fabricated to release aprotinin and thymosin β4 (Tβ4) in a programmable manner. The core part of the fiber is composed of hyaluronic acid and Tβ4, and the shell is PCL, which is further coated with heparin/gelatin/aprotinin to enhance biocompatibility. The *in vitro* assay demonstrates that the controlled release of aprotinin prevents initial excessive inflammation. The subsequent release of Tβ4 after 3 days induces the transition of macrophages from M1 into M2 polarization. The manipulation of inflammatory response further controls the expression of transforming growth factor-β and fibroblast activation, which oversee the quantity and quality of ECM remodeling. In addition, the gradual degradation of the scaffold allows cells to proliferate within the platform. *In vivo* implant evaluation convinces that PCL-based scaffolds possess the high capability to control the inflammatory response and restore the ECM to normal conditions. Hence, our work paves a new way to develop tissue engineering scaffolds for inflammation manipulation and ECM remodeling with peptide-mediated reactions.

## Graphical Abstract

**Figure:**
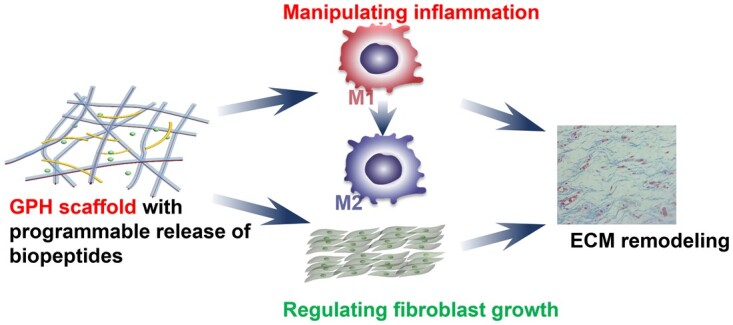

## Introduction

Tissue engineering scaffolds, acting as a temporary, artificial extracellular matrix (ECM), provide a physical and biological micro-environment for tissue repair and regeneration [1–3]. The successful tissue repair to restore the organ’s integrity mainly depends on ECM remodeling with the aid of scaffolds. Since ECM serves as a natural scaffold for cell organization into tissues and supports a dynamic microenvironment for signal exchanges with cytokine and growth factors manipulation, too little or too much ECM production can yield severe dysfunction [4, 5]. However, tissue engineering scaffolds are subject to the inflammatory response during implantation, and the host response to the implanted scaffolds remains a major challenge for ECM remodeling and tissue repair [6, 7].

Inflammatory response by macrophages is an unavoidable and essential host response for tissue regeneration. And macrophage plasticity governs the quality of ECM remodeling [8, 9]. Macrophage plasticity is reflected in the switching between pro-inflammatory M1 and anti-inflammatory, regeneration-promoted M2 phenotype [10–12]. It has been widely recognized that the prolonged presence of M1 macrophages aggravates inflammation and mediates transforming growth factor-β (TGF-β) overexpression in fibroblasts, resulting in excessive deposition of ECM and even fibrosis [13, 14]. Thus, promoting macrophages’ transformation from M1 to M2 phenotype is the dominant strategy to facilitate ECM remodeling and healthy tissue repair [15, 16]. However, due to the release of cytokines, metabolites and growth factors and the clearance of debris with macrophages, and the recruitment of fibroblasts to migrate, proliferate and produce new ECM by macrophages [17]. The arbitrary inhibition of M1 macrophages and premature promotion of the M2 phenotype are associated with delayed wound healing, excessive ECM deposition, poorly formed neovascularization and pathological fibrosis [18, 19]. Therefore, it is highly desirable to design the scaffold to control the delicate and temporally balance in these two phenotypic extremes to facilitate optimal ECM remodeling and tissue regeneration.

Electrospinning is a modern and robust technique for scaffold fabrication that mimics ECM to exchange signals with cells through the release of cytokine and growth factors [20]. Compared to the other techniques, electrospinning shows the unique advantage of encapsulating and modulating the release of multiple drugs in a programmable manner by varying the structure and composition of nanofibers [21]. Polypeptides have a relatively simple chemical structure, and mild storage conditions and can be obtained efficiently by solid-phase synthesis, which has been encapsulated in electrospun fibers for tissue engineering scaffolds [22, 23]. Thymosin β4 (Tβ4), can regulate angiogenesis and has anti-inflammatory and anti-fibrosis effects in tissue repair [24]. In addition, Tβ4 is reported to promote the polarization of M2 macrophages [25]. Aprotinin is another polypeptide that is widely used as an anti-inflammatory and anti-thrombotic drug in clinics. Aprotinin can inhibit macrophage activity and limit the binding of platelet p-selectin to inflammatory cells, thus effectively limiting the over-recruitment of inflammatory cells at the site of injury [26]. Thus, encapsulation of aprotinin and thymosin in the electrospun nanofibers and the controlled release of these drugs are expected to control the inflammation and ECM remodeling effectively. Gelatin is a partial hydrolytic product of collagen, which is an important component of ECM [27]. Heparin has special functions in anti-cell adhesion and anti-allergy [28]. The effect of foreign body reaction on tissue repair can be reduced by gelatin and heparin.

Here, we design polycaprolactone (PCL)-based core–shell structured fibers as the scaffold for ECM remodeling and tissue repair with electrospinning. The core part is composed of hyaluronic acid (HA) and Tβ4, and the shell part is PCL, which is further coated with heparin/gelatin/aprotinin. When the scaffold is implanted, aprotinin on the shell gradually releases and inhibits the over-recruitment of inflammatory cells on the surface of the scaffold to reduce excessive inflammation. After an inflammatory response for 3 days, Tβ4 is released from the fibers, inhibits the fusion of macrophages into multinucleated foreign body giant cells, and induces the transition from M1 into M2 polarization to facilitate ECM remodeling and tissue repair. During this process, the gelatin gradually degrades, which allows cells to proliferate within the scaffold. In addition, the coated heparin prevents ECM fibrosis around the scaffold. Therefore, through the programmable release of biological peptides from a PCL-based scaffold, the inflammation is well controlled and the normal ECM of damaged tissue is restored.

## Materials and methods

### Materials

Fetal bovine serum (FBS) and trypsin-EDTA are purchased from Sigma-Aldrich (St. Louis, MO, USA). Dulbecco’s modified Eagle’s medium (DMEM) and penicillin–streptomycin are purchased from Gibco Life Technologies (Grand Island, NY, USA). Tβ4 (Purity = 99.2%) and aprotinin (lyophilized powder, 3–8 TIU/mg solid, 95%) are purchased in Sigma-Aldrich (St. Louis, MO, USA), the antibody of CD86 (PE, IT2.2), CD206 (FITC, C068C2) are purchased from thermos Fisher Scientific (81WymanStreet, Waltham, MA, USA), PCL (Mw = 86000 Da), HA (40–100 KDa) and gelatin (Pharmaceutical grade, glue strength ∼240 bloom) are purchased in Energy Chemical (Shanghai, China). All cells are purchased from Beyotime Biotechnology of Shanghai. Dimethylformamide (DMF), trimethylamine, ethylenediamine, acrylamide, dichloromethane (DCM), tetrahydrofuran, acetone and xylene are reagent-grade products. Other reagents are AR-grade and used without further purification. Phosphate-buffered saline (PBS 0.9% NaCl, 0.01 M phosphate buffer, pH 7.4) is prepared freshly.

### Preparation of scaffold with electrospinning

The electrospinning process has been reported elsewhere [29, 30]. In a 15 kV high-voltage field, aluminum foil in flat plate receiver at rest is used to receive the ejected fibers. A precision pump (Smith medical Longer Precision Pump WZS-50F6, China) is used to control the injection rate with 1 ml/h. The single injection method is adopted. For the electrospun solution, HA aqueous solution with a concentration of 1.2 wt% is prepared. Then, Tβ4 solution (Tβ4, 3.33 mg/ml) is mixed with HA solution to obtain 1% HA-Tβ4 hydrosol. Afterward, 3.03 ml DCM, 0.01 g Span-80, and 1% HA-Tβ4 hydrosol (150 μl) are mixed at high speed to obtain water-in-oil (W/O) emulsions containing uniformly micro-sol particles. At last, 0.5 g PCL and 2.11 ml DMF are dissolved in the water-in-oil (W/O) emulsions to obtain PCL/HA-Tβ4 (PH) electrospun solution. Mixing 0.5 g PCL, 3.03 ml DCM and 2.11 ml DMF to get the PCL electrospun solution as a control. After obtaining the PH fiber, the fiber membrane is immersed in 10 ml 10% ethanol/water solution containing 1 g gelatin, 0.1 g heparin sodium and 25 mg aprotinin for 5 min. Due to the three-dimensional structure of the scaffold, the solution can easily soak into the scaffold and absorb on the fiber surface. Then the heparin and gelatin in the scaffold are cross-linked in glutaraldehyde steam for 2 h after lyophilization to obtain the GPH fiber membrane. Then the fibers are cross-linked in glutaraldehyde steam for 2 h after lyophilization to obtain the GPH fiber membrane.

### Characteristics of the scaffold

The morphology is observed using a scanning electron microscope (SEM, Sirion-100, FEI, USA) at a voltage of 15 kV to observe the microstructural morphology of the scaffold. The average diameter of the sample is analyzed by measuring 100 random fibers with Image J software. The core–shell structure of PH fiber is verified by TEM (TEM JEM1400, Japan). The different scaffolds are analyzed by infrared spectroscopy (FTIR, BRUKER, VERTEX 80/80v, Germany) to verify the integration of different components. The surface wettability is evaluated by the sessile drop method with a pure water droplet (ca. 2 μl) 5 s after dripping using a contact angle goniometer (DSA, KRUSS GMBH, Germany). The mechanical properties of the scaffolds are tested by a mechanical testing machine.

### Thymosin β4 and aprotinin release

Microfibers with a size of 1 × 1 cm (100 mg) are immersed in PBS solvent (10 ml). Then, at the desired time from 1 to 9 days, the concentration of Tβ4 and aprotinin in the solvent are monitored with Tβ4 and aprotinin ELISA kit.

### The degradation of the scaffold

An accelerated *in vitro* degradation method is considered to evaluate the degradability of all scaffolds [31]. In brief, the scaffolds are cut into 10 * 10 * 0.3 mm small blocks and are immersed in sodium hydroxide solution (5 M, pH = 14.17). The samples are removed every hour, rinsed with deionized water and dried. The degradation of the scaffold is calculated according to the following equation:

(1) $$\mathrm{Degradation}\;\left(\%\right)=\frac{M\left(t\right)}{M\left(0\right)}*100\%\;,$$

where *M*(*t*) is the residual mass of the scaffold after *t* hour of degradation and *M*(0) is the initial mass of the scaffold.

### Protein adsorption experiment

Bovine serum albumin (BSA) is used as a model protein to evaluate the protein adsorption on the scaffold. After being equilibrated with PBS overnight, the specimens (1 × 1 cm) are moved into PBS solution containing BSA (0.1 mg/ml) at 37°C for 2 h. Then each sample is rinsed by gentle shaking in the new PBS solution. Subsequently, the samples are immersed in 1 ml of PBS solution containing 1 wt% of sodium dodecyl sulfate (SDS), and the protein adsorbed on the surface is entirely desorbed by sonication for 20 min. A micro bicinchoninic acid (BCA) protein assay reagent kit based on the BCA method was used to determine the protein concentration in the SDS solution. The concentrations were determined using TECAN (GENIOS, Austria) operating at 562 nm.

### The blood test

The hemolysis rate and coagulation index of different samples are studied according to previous work [32]. The samples (1 × 1 cm^2^) are incubated with red blood cells (RBCs) for 2 h at 37°C. The RBCs in PBS and water are set as a negative and positive control, respectively. The RBCs are removed by centrifugation (3000 rpm, 5 min), the supernatant is transferred to 96-well plates, and its absorbance (OD) at 541 nm is measured using a microplate reader. The hemolysis ratio (HR) is calculated according to the following formula:

(2) $$\mathrm{Hemolysis}\;\mathrm{rate}\;\left(\%\right)=\frac{{\mathrm{OD}}_{\mathrm{sample}}-{\mathrm{OD}}_{\mathrm{neg}}}{{\mathrm{OD}}_{\mathrm{pos}}-{\mathrm{OD}}_{\mathrm{neg}}}\times 100\%.$$

The whole blood (200 μl) is dropped on the surface of a fiber membrane, and then 20 μl of 0.2 M CaCl_2_ solution is added to the blood to activate clotting. The control group is produced by dropping blood on the glass. Each sample is incubated for 5 min at 37°C. Next, distilled water (25 ml) is carefully added to the beaker and set at 37°C with shaking at 30 rpm for 10 min. The absorbance of fresh citrated whole blood in deionized water is used as contrast (blank). Finally, the blood clotting index (BCI) of nanofibers is calculated from the following equation:

(3) $$\mathrm{BCI}\;(\%)=\frac{{\mathrm{OD}}_{\mathrm{sample}}}{{\mathrm{OD}}_{\mathrm{blank}}}\times 100\%.$$

OD_sample_ is the absorbance of the test samples, and OD_blank_ is the absorbance of the contrast group.

The BCI in 15, 25, 35, 45 and 55 min are also researched similarly. The PT and APTT are measured by PT and APTT assay kit (Shanghai Sun Biotechnology Co., Ltd).

### Cell experiment

To verify the effects of samples on cells at different times, the extracts of each sample in PBS are taken to detect the effects of extracts on different cells [33]. Briefly, 1.0 × 10^4^ L929 cells are seeded into a 96-well plate. After incubation with 5% CO_2_ for 24 h at 37°C, the culture medium is renewed by 100 μl medium (DMEM) supplemented with 10% FBS. Five percent concentrations of sample extract are added to cultural medium for incubation in 24 h. Then 10 μl of CCK-8 is added to each well, followed by incubation for 2 h. Finally, the microplate reader is used to measure the absorbance at 450 nm. The cell viability is calculated according to the following formula:

(4) $$\mathrm{Cell}\;\mathrm{viability}\;(\%)=\frac{{\mathrm{OD}}_{\mathrm{sample}}}{{\mathrm{OD}}_{\mathrm{contrast}}}\times 100\%.$$

OD_sample_ is the absorbance of the test samples, and OD_contrast_ is the absorbance of the contrast group. Farther, L929 cells are stained with calcein and observed by confocal microscopy.

To verify the regulation effect of fiber on inflammation in different periods, different extracts (extract in 2 and 7 days, 5 wt%) are used to treat macrophages treated by 100 ng/ml LPS for 6 h. After incubating at 37°C for 24 h, cytokines TGF-β, TNF-α and IL-10 in the cell culture medium are detected. Besides, CD206 and CD86 are used to mark M2 macrophages to verify the polarization of macrophages.

### *In vivo* tissue regeneration study

All animal procedures are performed by the Guidelines for Care and Use of Laboratory Animals of Changchun Institute of Applied Chemistry (Chinese Academy of Sciences) and approved by the Animal Ethics Committee of Changchun Institute of Applied Chemistry (Chinese Academy of Sciences). For each experimental group, three female Kunming mice at 6 weeks of age (about 30 g) are used and all mice are randomly selected. Control the temperature at about 20°C.

To evaluate the effectiveness of GPH fibers in ECM remodeling, we construct a subcutaneous injury model. Briefly, the skin of the mice is cut open to expose the subcutaneous tissue. A part of the subcutaneous tissue is cutoff, and different scaffolds are placed in the defect. Suturing the skin. The tissues around the scaffold are removed and analyzed at 2 and 6 weeks. These tissues are treated with H&E staining and Masson staining respectively. All image data are quantified by Image J software.

### Statistical analysis

The BCI, HR, TGF-β, IL-10, and TNF-α are given as means ± standard deviation. Statistical analysis was performed using Origin software, with *post hoc* analysis by Bonferroni’s multiple comparison test when appropriate. Differences are considered statistically significant at *P* ≤ 0.05.

## Results and discussion

**Scheme 1.**
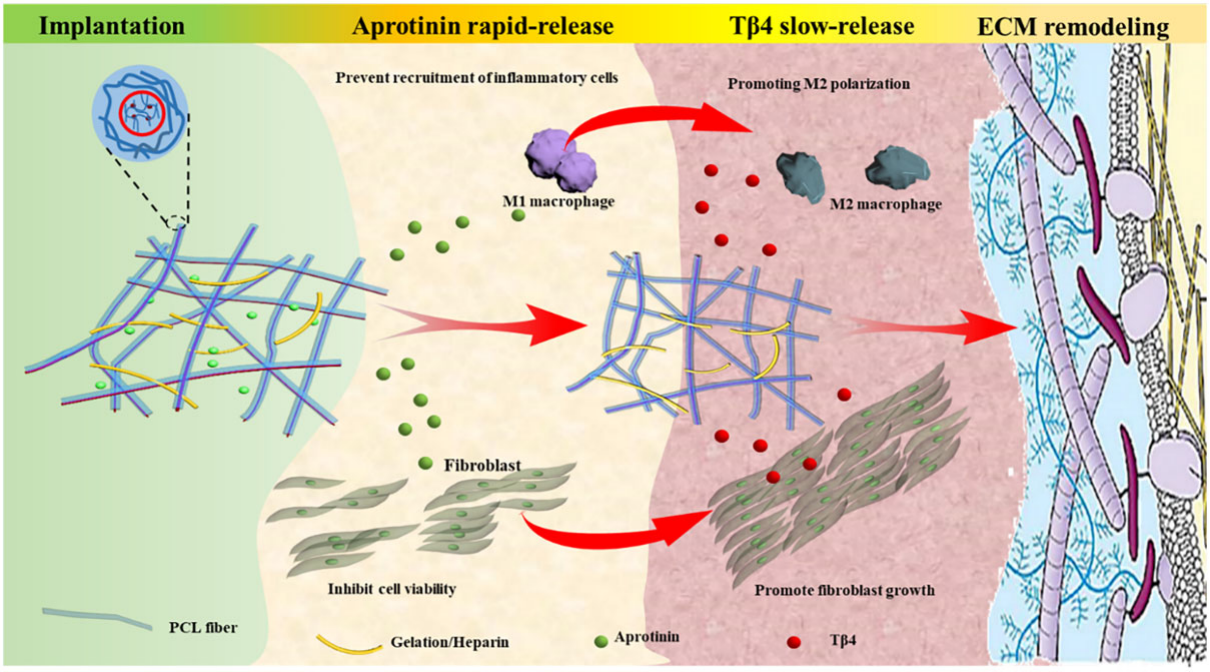
Bioactive fibrous scaffolds with programmable release of polypeptides regulate inflammation and extracellular matrix remodeling.

Inflammation and ECM dysfunction caused by implanted interventional materials bring great challenges to the application of tissue-engineered scaffolds. To overcome this problem, we fabricate a PCL-based scaffold (GPH) that releases the aprotinin and Tβ4 in a programmable manner to control in the inflammatory response and further adjust the expression of TGF-β and fibroblast activation resulting in normal ECM remodeling and healthy tissue repair. (Scheme 1). Rapid release of aprotinin outside the fibers inhibits early inflammation and prevents over-recruitment of inflammatory cells, which prevents excessive collagen deposition during the repair. The delayed release of Tβ4 in the fibers promotes the polarization of macrophage M2 and accelerates repair.

### Construction of multicomponent composite fiber scaffold

Multicomponent composite fiber scaffolds (GPH) are constructed by combined electrospinning and freeze-drying technology. The inner fiber is composed of core–shell structured PCL/HA/Tβ4 microfibers with PCL as the shell and HA-Tβ4 as the core. The outside consists of heparin/gelatin/aprotinin, which inhibits early inflammation and prevents the over-recruitment of inflammatory cells. For comparison, PCL fibers and PH fibers are prepared by conventional electrospinning.

**Figure 1.**
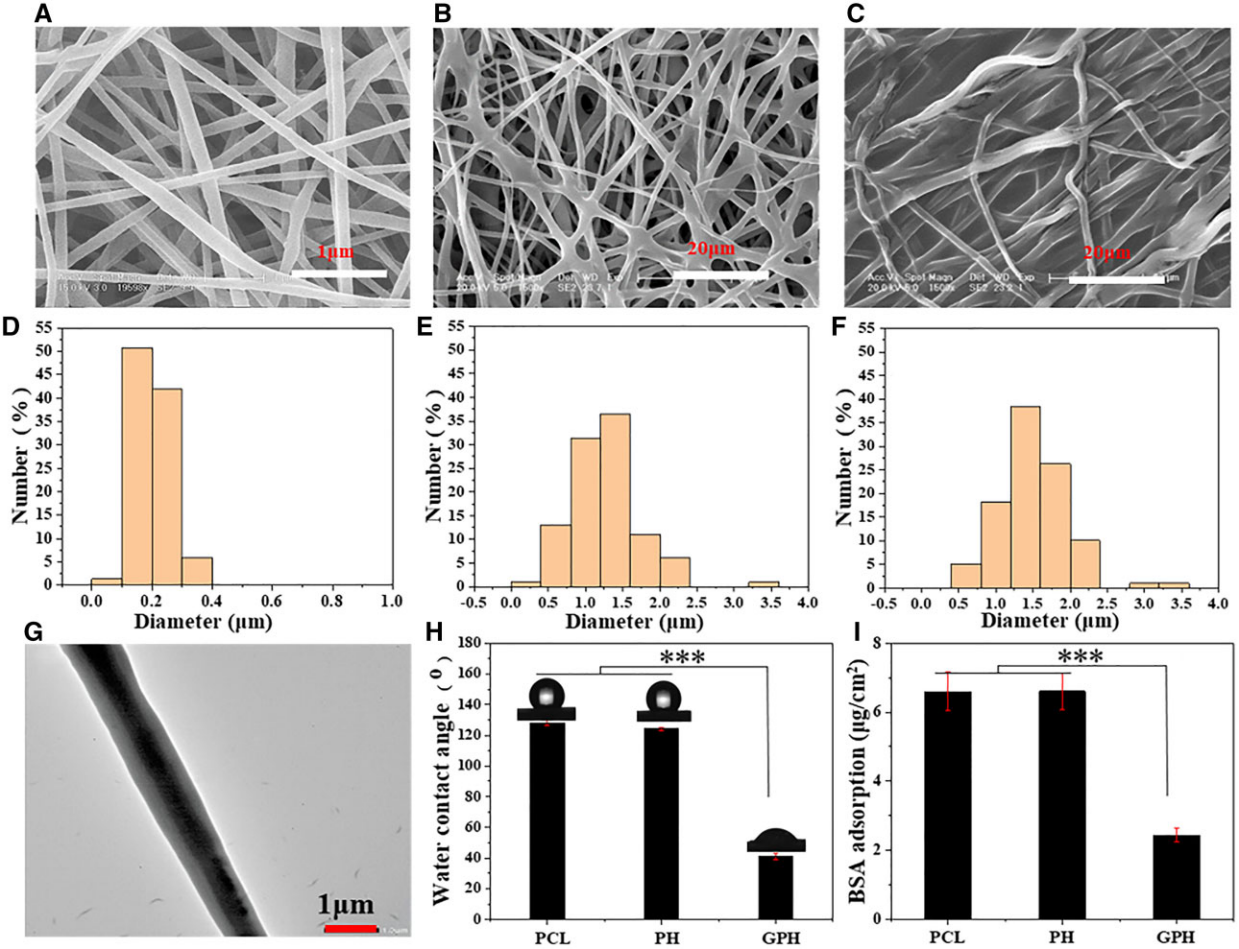
Construction of multicomponent composite fiber scaffold. (**A**–**C**) SEM images of PCL, PH and GPH fiber scaffold. (**D**–**F**) Diameter distribution of PCL, PH and GPH fiber scaffold. (**G**) TEM image of core–shell fibers. (**H**) The water contact angle, of PCL, PH and GPH fiber scaffold. (**I**) Adsorption capacity of BSA protein. The data are shown as the means ± standard deviation from three independent experiments. ***P < 0.001 indicates significant differences between the indicated columns.

Neat PCL microfibers distribute uniformly with a diameter of about 200 nm (Fig. 1A and D). The diameter and dispersion of PH fibers are significantly increased because of emulsion spinning from Fig. 1B and E. The fiber diameter of GPH is about 1.5 μm due to polymer (such as gelatin) coating on the fiber surface (Fig. 1C and F). And the TEM image of PH fibers shows that the core–shell structure fiber is obtained successfully (Fig. 1G). Infrared characterization also confirm the loading of each component in the GPH (Supplementary Fig. S1). Gelatin degrades rapidly in the body, which allows cells to proliferate within the scaffold [34]. Compared with PH and PCL, GPH is more hydrophilic (Fig. 1H). Compared with PCL and PH, the protein adsorption capacity of GPH scaffold is lower (Fig. 1I). These features of the GPH scaffold can reduce the protein adsorption of the material after implantation, thus reducing the inflammatory response [35, 36]. Besides, GPH has excellent mechanical properties (Supplementary Fig. S2), which allows the scaffold to be used in tissue engineering.

### The degradation of scaffold

PCL has attracted more and more attention because of its excellent biocompatibility and biodegradability [35]. However, the degradation rate of PCL is slow under physiological conditions (Supplementary Fig. S3). And microstructure of different scaffolds after soaked in PBS for 6 weeks as shown in Supplementary Fig. S4, PCL and PH scaffold do not change obviously, but heparin and gelatin clearly covered the whole surface of the scaffold in GPH group. The degradation process of PCL is the hydrolysis of the ester bond, and the degradation mechanism of the ester bond under alkaline conditions is as follows [37, 38]:
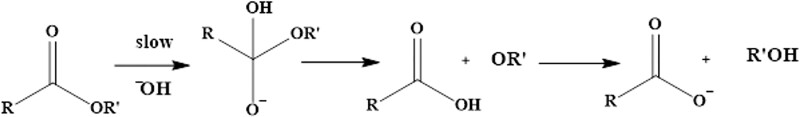


The first step of the mechanism diagram determines the rate of ester hydrolysis. Therefore, the degradation rate of PCL can be adjusted by adjusting the system’s pH. To understand the whole process of scaffold degradation, the scaffold is immersed in NaOH solution (pH = 14.17) to accelerate the degradation.

**Figure 2.**
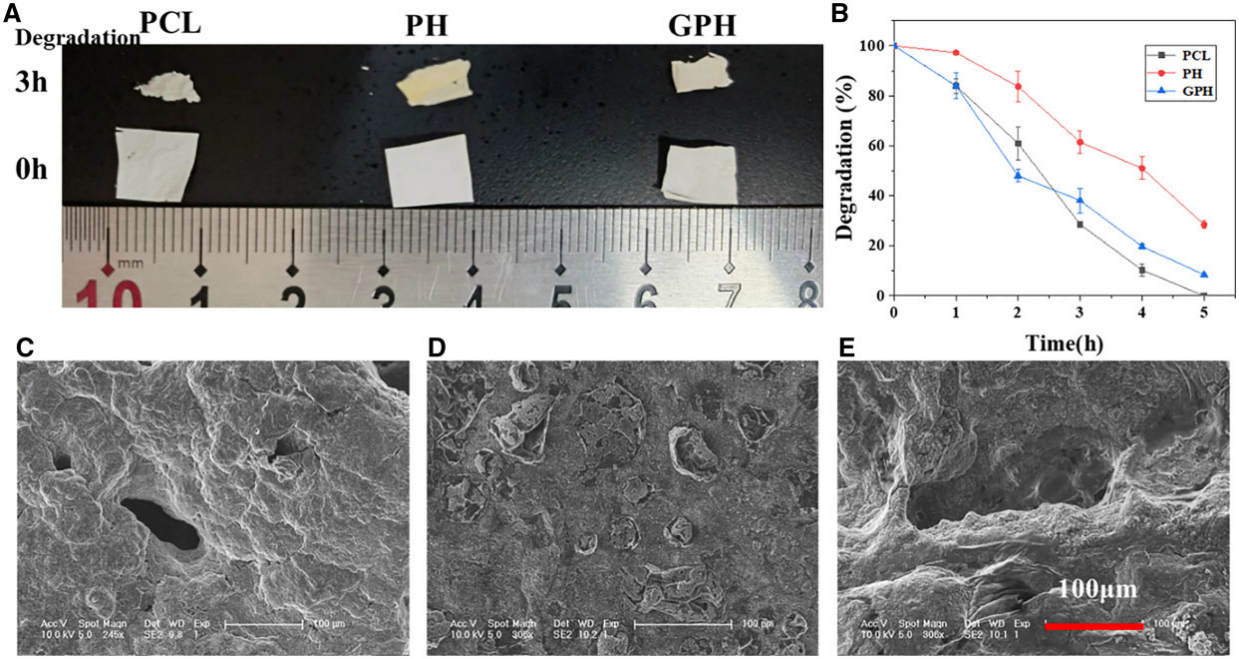
The degradation of the scaffold. (**A**) The image of PCL, PH and GPH in 0 h and soaked in NaOH solution (pH = 14.17) for 3 h. (**B**) The curve of scaffold degradation rate with time. (**C**–**E**) The SEM images of PCL, PH and GPH scaffold after soaking in the NaOH solution for 3 h. The data are shown as the means ± standard deviation from three independent experiments.

The image of PCL, PH and GPH in 0 h and soaked in the NaOH solution for 3 h is shown in Fig. 2A. All scaffolds are partially degraded. From the curve of scaffold degradation (Fig. 2B), the PH degradation rate is significantly slower than PCL, which may be due to the larger fiber diameter of PH [39]. And in the first 2 h, the GPH has the fastest degradation rate because of the degradation of gelatin. The degradation of gelatin causes large pores and surface areas, promoting cell infiltration [34]. From the SEM image of the scaffold after soaking in the NaOH solution for 3 h (Fig. 2C–E), the structure of PCL and GPH change considerably. While the PH keeps the morphology of the membrane, a large number of voids appear. All these pieces of evidence indicate that GPH scaffolds have better degradability.

### GPH regulates fibroblast proliferation

**Figure 3.**
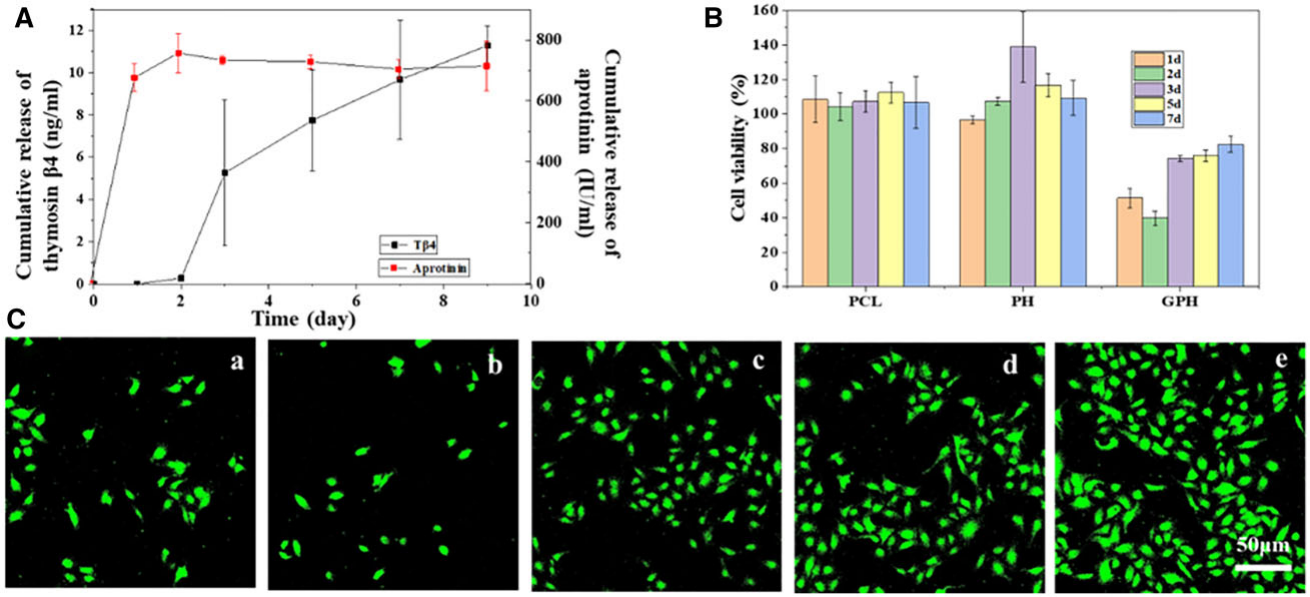
GPH fiber scaffold regulates L929 cell proliferation. (**A**) Release curve of Tβ4 and aprotinin from GPH fiber scaffold. (**B**) Effects of extracts (the extracts concentration is 5% in cell culture medium) at a different time of PCL, PH and GPH scaffold to fibroblast cell (L929) proliferation. (**C**) Laser confocal images of L929 cells incubated with extracts of GPH scaffold (the extract concentration is 5% in cell culture medium) at different times for 24 h. **a**, 1 day; **b**, 2 days; **c**, 3 days; **d**, 5 days; **e**, 7 days. Fluorescently labeled cells represent viable cells. The data are shown as the means ± standard deviation from three independent experiments.

GPH fiber scaffold is externally loaded with aprotinin, which inhibits cell viability and acute inflammation and prevents excessive recruitment of inflammatory cells. The fiber core is packed with Tβ4, which can promote the M2 polarization of macrophage and accelerate the repair. Figure 3A shows the release of aprotinin and Tβ4 in 9 days. Aprotinin is released almost entirely during 1 day, while Tβ4 is released from the third day and can continuously be removed for a long time. Rapid release of aprotinin can treat acute inflammation. While the delayed release of Tβ4 can promote L929 cell proliferation and M2 polarization of macrophage to accelerate standard ECM remodeling. The drug release program is designed to maintain the inflammatory balance, accelerate the repair at the scaffold area and prevent tissue fibrosis [40].

Fibroblasts can secrete various ECM proteins, such as collagen and cytokines-regulated ECM, are the predominant producer of ECM across all organs [4]. Persistent fibroblast activities can lead to tissue fibrosis, but excessive inhibition of fibroblast growth is not conducive to ECM remodeling [41, 42]. Here, we verify the effect of scaffold on fibroblast growth at different times. Five percent concentration extract of scaffolds at various times is used to incubate with L929 cells to explore the impact of the scaffold on cell activity. With the extended scaffold extraction time, PCL extract has no significant effect on cell growth, while PH extract can promote cell proliferation. However, GPH extracts show the impact of inhibiting cell proliferation and then promoting cell proliferation (Fig. 3B). The results are consistent with the curve of drug release. Laser confocal images of L929 cells incubated with 5% extracts at different times of GPH also indicate the regulation of scaffold on L929 cell growth (Fig. 3C). Therefore, GPH may regulate cell growth through the programmed release of drugs, which will ensure the remodeling of the ECM without fibrosis. This provides a new idea for preventing tissue hyperplasia and fibrosis in the tissue engineering field. The effects of scaffold on blood are also investigated (Supplementary Fig. S5). High anti-protein adsorption, high hemocompatibility, and good anticoagulation ability mean that GPH scaffold has the potential to be used in tissue engineering, even vascular tissue engineering.

### GPH fiber scaffold regulates the polarization of macrophages

**Figure 4.**
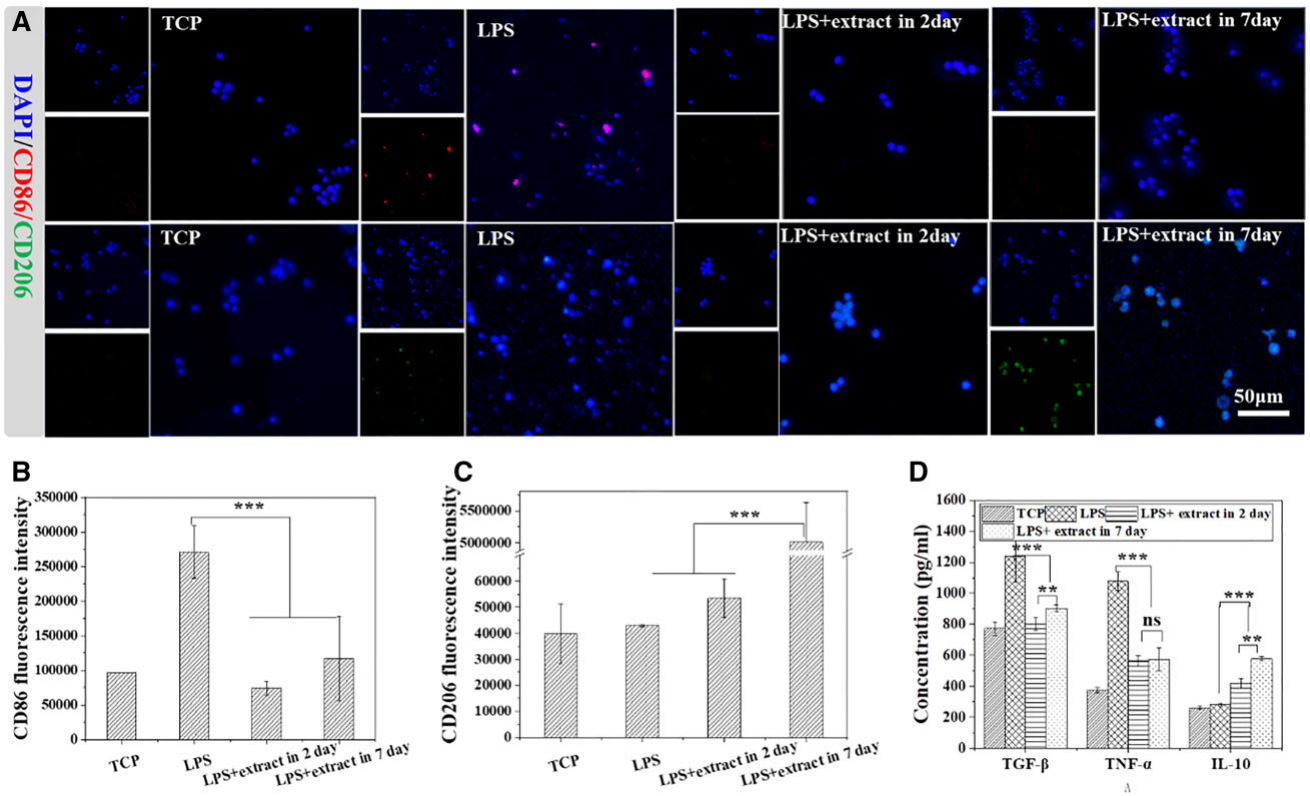
GPH fiber regulates the polarization of macrophages. (**A**) Confocal laser images of macrophages after different treatments with LPS, extract in 2 days and extract in 7 days. (**B** and **C**) Quantization of CD86 and CD 206 fluorescence intensity in confocal. (**D**) The concentration of TGF-β, TNF-α and IL-10 in cell supernatant of different groups. The data are shown as the means ± standard deviation from three independent experiments. **P < 0.01, ***P < 0.001 indicates significant differences between the indicated columns.

Inflammatory cells quickly migrate to the site of inflammation within a few hours, aggravating the inflammation [43]. Macrophages form multinucleated giant cells 3–9 days after the onset of rash, leading to foreign body response and fibrosis [20]. The trend in drug release of the GPH scaffold matches precisely with the inflammatory process. To verify the regulation of the GPH scaffold on inflammation at different stages, the extracts in 2 and 7 days of GPH scaffold are co-incubated with LPS-activated macrophages, and the polarization of M1 and M2 of macrophages is detected. As shown in Fig. 4A, the extract (both in 2 days and in 7 days) significantly inhibit the M1 polarization of LPS-stimulated macrophage and inhibits the inflammatory response. Quantification of CD86 (marker of M1 macrophage) fluorescence intensity and the concentration of TNF-α (one of the pro-inflammatory factors) show the same results (Fig. 4B and D). This demonstrates that the GPH scaffold can maintain anti-inflammatory properties for a long time. Then, M2 macrophages are characterized by CD206 (marker of M2 macrophage). In Fig. 4A and C, the extract in 7 days can promote M2 macrophages, but the second day extract does not. The result of IL-10 is the same as the confocal results (Fig. 4D). These results indicate that GPH can maintain inflammatory balance by inhibiting macrophage activity in the early stage and promoting M2 polarization in the later stage. Maintaining inflammatory balance and promoting M2 polarization of macrophage are important in tissue engineering, which is one of the keys to preventing tissue fibrosis [44]. This result is consistent with the trend of drug release. The manipulation of inflammatory response further controls the expression of TGF-β and fibroblast activation, which guide the quantity and quality of ECM remodeling. TGF-β is mainly expressed by macrophages and plays a huge role in ECM repair. Insufficient TGF-β secretion is not conducive to the repair of ECM while excessive TGF-β secretion may cause fibrosis [17]. So under the condition of inflammation, the effect of scaffold on TGF-β secretion of macrophage at different time is studied. As shown in Fig. 4D, TGF-β overexpression in inflammatory conditions may lead to fibrosis. And TGF-β expression is significantly inhibited by the extracts on the second day, and has a certain rise on the seventh day. The trend favors inhibition of fibrosis and promotion of ECM repair.

### GPH fiber scaffold inhibits fibrosis during tissue repair

**Figure 5.**
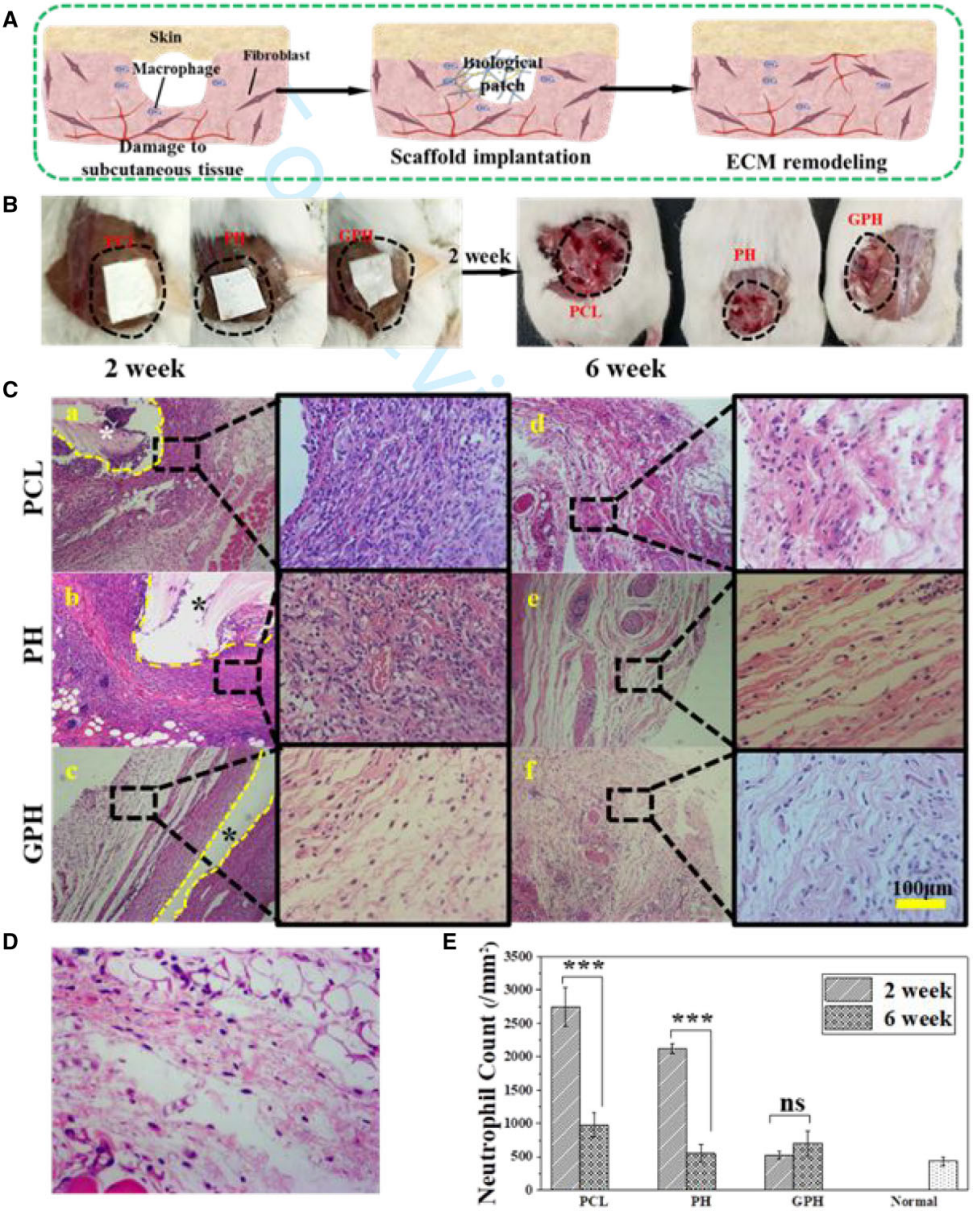
GPH fiber scaffold inhibits fibrosis during tissue repair. (**A**) The diagram of an *in vivo* experiment. (**B**) Left is the image of the scaffold implantation into the injury site, and right is an image of the implantation position in 2 weeks after removing the scaffold. (**C**) The H&E staining of PCL, PH and GPH group in 2 weeks (**a**–**c**) and 6 weeks (**d**–**f**). *Indicates the location of the scaffolds. (**D**) The H&E staining of normal tissue. (**E**) Quantification of neutrophils in different groups. The data are shown as the means ± standard deviation from three independent experiments. ***P < 0.001 indicates significant differences between the indicated columns.

To evaluate the tissue repair during long-term implantation, different scaffolds are implanted at the injury site under the skin of the Kunming mouse for 6 weeks (Fig. 5A). The image of the wound after scaffold implantation is shown in Fig. 5B. The images of the mouse after the scaffold are removed in 2 weeks show that the PCL group and PH group appear obvious ulceration and redness, but the GPH group showed no obvious abnormalities, which proves the anti-inflammation capacity of GPH (Fig. 5B). The scaffold and adjacent tissues are removed at 2 and 6 weeks. The tissues are stained with H&E and Masson, respectively. From the H&E staining in Fig. 5C, there is a significant decrease in inflammation in each group from the second week to the sixth week. And the GPH scaffold shows the best anti-inflammatory effect compared with PCL and PH in 2 weeks. The result indicates that the GPH can minimize early inflammation. The inflammatory reaction in the PH group and GPH group has completely subsided because of the loaded of Tβ4, but the PCL group still maintains severe inflammation compared to normal tissue (Fig. 5D) in 6 weeks. All these results can also be demonstrated in the quantitative of inflammatory cells (Fig. 5E). So the GPH scaffold can maintain the balance of inflammatory response in tissue repair.

**Figure 6.**
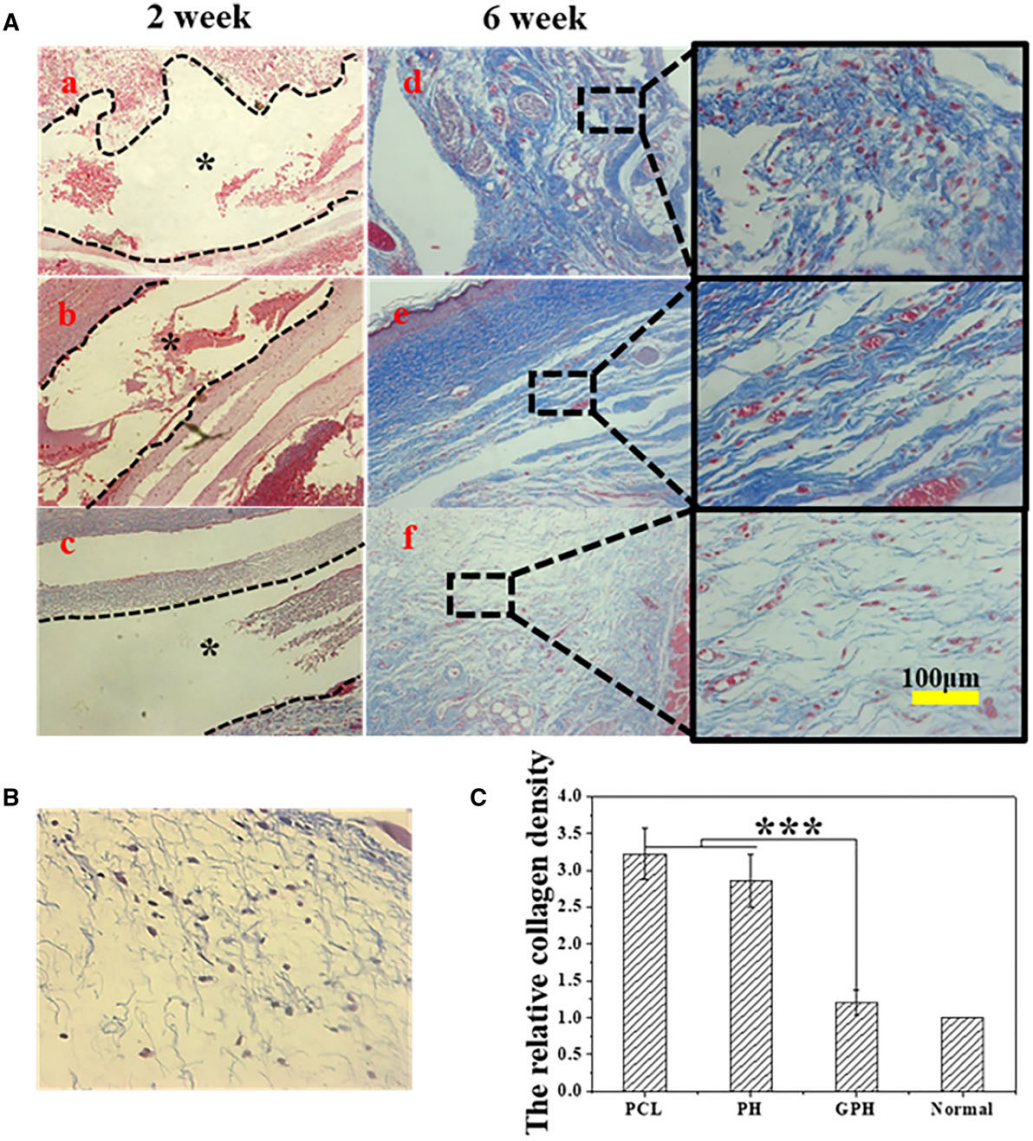
GPH fiber scaffold inhibits fibrosis during tissue repair. (**A**) The Masson staining of PCL, PH and GPH group in 2 weeks (**a**–**c**) and 6 weeks (**d**–**f**). (**B**) The Masson staining of normal tissue. (**C**) The relative collagen density in different groups in 6 weeks. The collagen density in the normal group is considered 1. The data are shown as the means ± standard deviation from three independent experiments. ***P < 0.001 indicates significant differences between the indicated columns.

The collagen structure of the tissue is characterized by Masson staining [45]. With the degradation of the scaffold, the ECM of tissue is gradually reconstructed. Each group has no collagen deposition at 2 weeks. Compared with the GPH group, tissues of the PCL and PH group have serious fibrosis (Fig. 6A) in 6 weeks, which suggests that inhibiting early inflammation can alleviate tissue fibrosis. In addition, there are a lot of gaps in the nascent ECM in the PCL group. The collagen density of the PH group is higher than that of the GPH group and normal tissue (Fig. 6B and C). The fiber arrangement of the PH group is very regular, this means that collagen I is overexpressed in the PH group, which is the main feature of scar tissue [42]. While the fiber arrangement of GPH groups is relatively chaotic. The collagen density and construction of the GPH group are similar to normal tissue. Therefore, in the field of tissue-engineered scaffolds, maintaining inflammatory balance by limiting early inflammation and promoting M2 polarization of macrophages can effectively prevent regeneration tissue fibrosis and accelerate the ECM repair [46].

## Conclusion

In summary, a PCL-based scaffold with core–shell structured electrospun nanofibers is fabricated for the programmable release of aprotinin and Tβ4. The core part of the fiber is composed of HA and Tβ4, and the shell is PCL, which is further coated with heparin/gelatin/aprotinin to enhance biocompatibility. The *in vitro* assay demonstrates that the control release of aprotinin prevents initial excessive inflammation, and the subsequent release of Tβ4 after 3 days induces the transition of macrophages from M1 into M2 polarization. The manipulation of inflammatory response further controls the expression of TGF-β and fibroblast activation, which guide the quantity and quality of ECM remodeling. In addition, the gradual degradation of the scaffold promotes cell infiltration. *In vivo* implant evaluation convince that PCL-based scaffolds possess the high capability to control the inflammatory response and to restore the ECM to normal conditions. Hence, our work paves a new way to develop tissue engineering scaffolds for inflammation manipulation and ECM remodeling with programmable delivery of polypeptides.

## Supplementary Material

rbad010_Supplementary_Data
