# Supplementary material for: Bioactive fibrous scaffolds with programmable release of polypeptides regulate inflammation and extracellular matrix remodeling
Source: Regen Biomater. 2023 Feb 20;10:rbad010. doi: 10.1093/rb/rbad010 (PMC9960911; doi:10.1093/rb/rbad010)
Supplement: rbad010_Supplementary_Data [file rbad010_supplementary_data.docx]

**Supporting Information**

**Bioactive Fibrous Scaffolds with Programmable Release of Polypeptides Regulate Inflammation and Extracellular Matrix Remodeling**

*Zehong Xiang,^1,2^* *Xinghua Guan,^1,2^ Zhifang Ma,*^,1^ Qiang Shi,*^,1,2,3^ Mikhail Panteleev ^4,5^*

*Fazly I. Ataullakhanov,^4,5^*

*^1^*^.^ State Key Laboratory of Polymer Physics and Chemistry, Changchun Institute of Applied Chemistry, Chinese Academy of Sciences, Changchun, Jilin 130022, China

*^2^*^.^ University of Science and Technology of China, Hefei, Anhui, 230026, China

*^3^*^.^ Key Laboratory of Polymeric Materials Design and Synthesis for Biomedical Function, Soochow University, Suzhou, 215123, China

*^4.^*Dmitry Rogachev Natl Res Ctr Pediat Hematol Oncol, 1 Samory Mashela St, Moscow, 117198, Russia.

*^5.^* Faculty of Physics, Lomonosov Moscow State University, Leninskie Gory, 1, build. 2, GSP-1, Moscow 119991, Russia

**Corresponding Authors**

Tel: +86 431 85262388. Fax: +86 431 85262126.

E-mail: shiqiang@ciac.ac.cn

**1. Infrared spectrum characterization of gelatin, heparin, PCL,PH and GPH.**

From Figure S1, the characteristic peak of PCL, heparin and gelatin can be found in the infrared of GPH, demonstrating the successful loading of each component in the GPH scaffold.


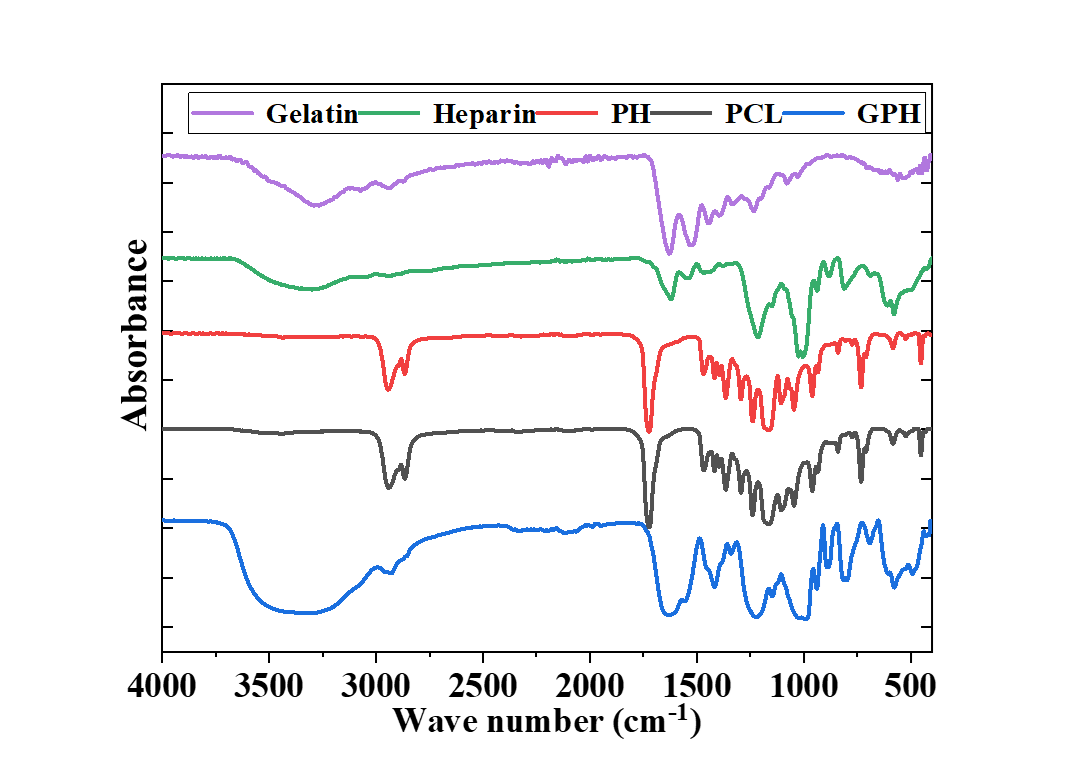


**Figure S1.** Infrared spectrum characterization of gelatin, heparin, PCL, PH and GPH.

**2. Mechanical properties characterization of different scaffolds.**

Compared with PCL scaffolds, the elastic modulus, yield strength and elongation at break of PH scaffolds and GPH scaffolds are significantly improved.


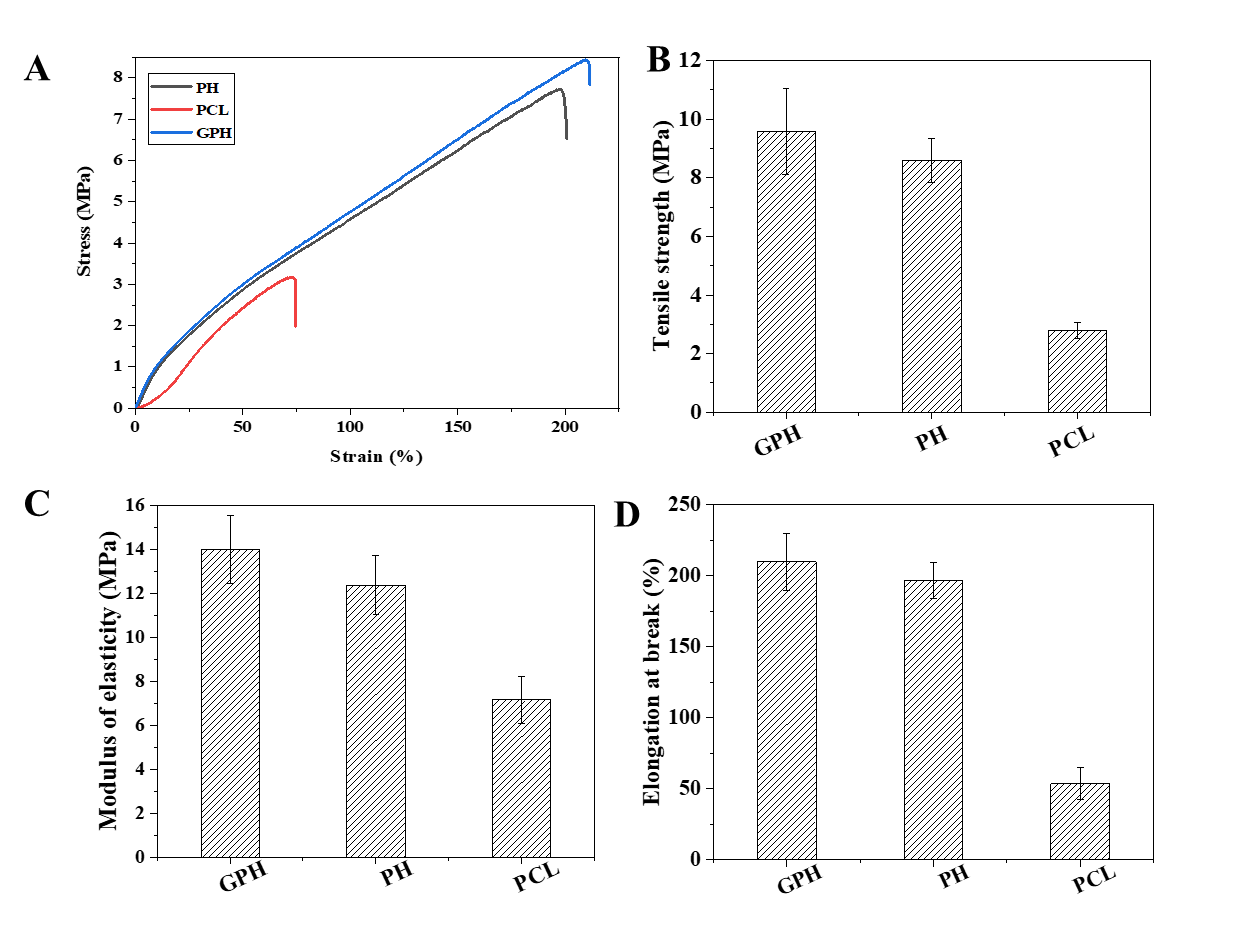


**Figure S2.** Mechanical properties characterization of PCL, PH and GPH. (A) Stress - strain curves of different supports. (B), (C) and (D) represent respectively the tensile strength, elasticity modulus and elongation at break.

**3.** **Degradation of scaffolds in PBS**

Different fiber scaffolds are immersed in PBS to study their degradation under physiological conditions. In brief, the scaffolds are cut into 10*10*0.3 mm small blocks and are immersed in PBS solution. The samples are removed every week, rinsed with deionized water, and dried. The degradation of the scaffold is calculated according to the following equation:

$Degradation \left( \% \right)=\frac{M\left( t \right)}{M\left( 0 \right)}*100\%$(1)

Where M(t) is the residual mass of the scaffold after t hour of degradation and M(0) is the initial mass of the scaffold.


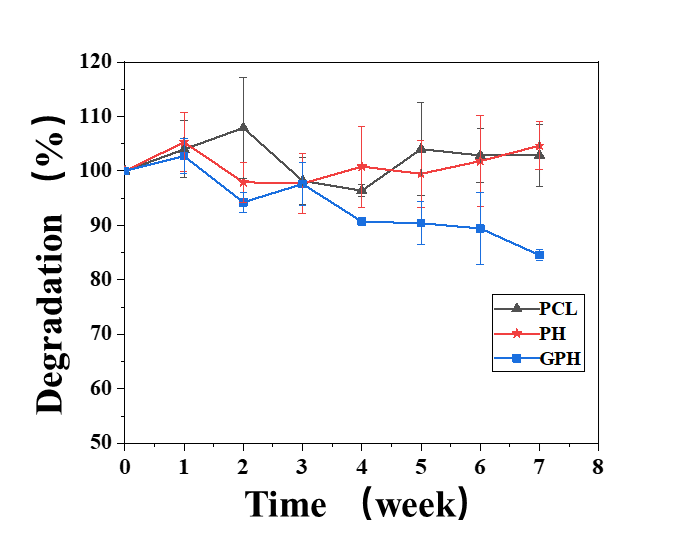


**Figure S3**. Degradation curves of PCL, PH and GPH scaffolds in PBS at 6 weeks.


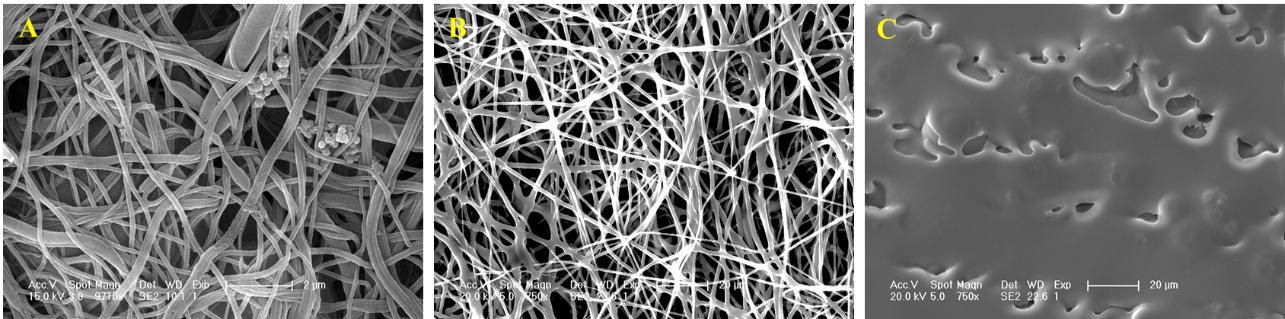


**Figure S4**. SEM image of PCL, PH and GPH scaffolds in PBS at 6 weeks.

**4. Blood compatibility and anticoagulant ability of GPH fiber scaffold.**

Protein adsorption is an important factor of coagulation and inflammation caused by medical materials. Reducing protein adsorption can effectively reduce the adverse reactions of materials. Figure S4A shows that the hemolysis rate of all samples is lower than 2%, indicating these fibers have no toxic effect to red blood cells. Heparin sodium exhibits the highest BCI with excellent anticoagulant capability due to efficient binding of AT-III to inhibit thrombus. Due to the load of heparin in the GPH scaffold, the anticoagulant ability of the scaffold was studied. The APTT (Figure S4B), PT (Figure S4C) and BCI (Figure S4D) results demonstrate GPH has high antithrombotic capability. The coagulation index of the scaffolds at different time are further investigated, which indicated that the GPH scaffold has long-term anticoagulant ability (Figure S4E). High anti-protein adsorption, high hemocompatibility and good anticoagulation ability mean that GPH scaffold has the potential to be used in tissue engineering even vascular tissue engineering.


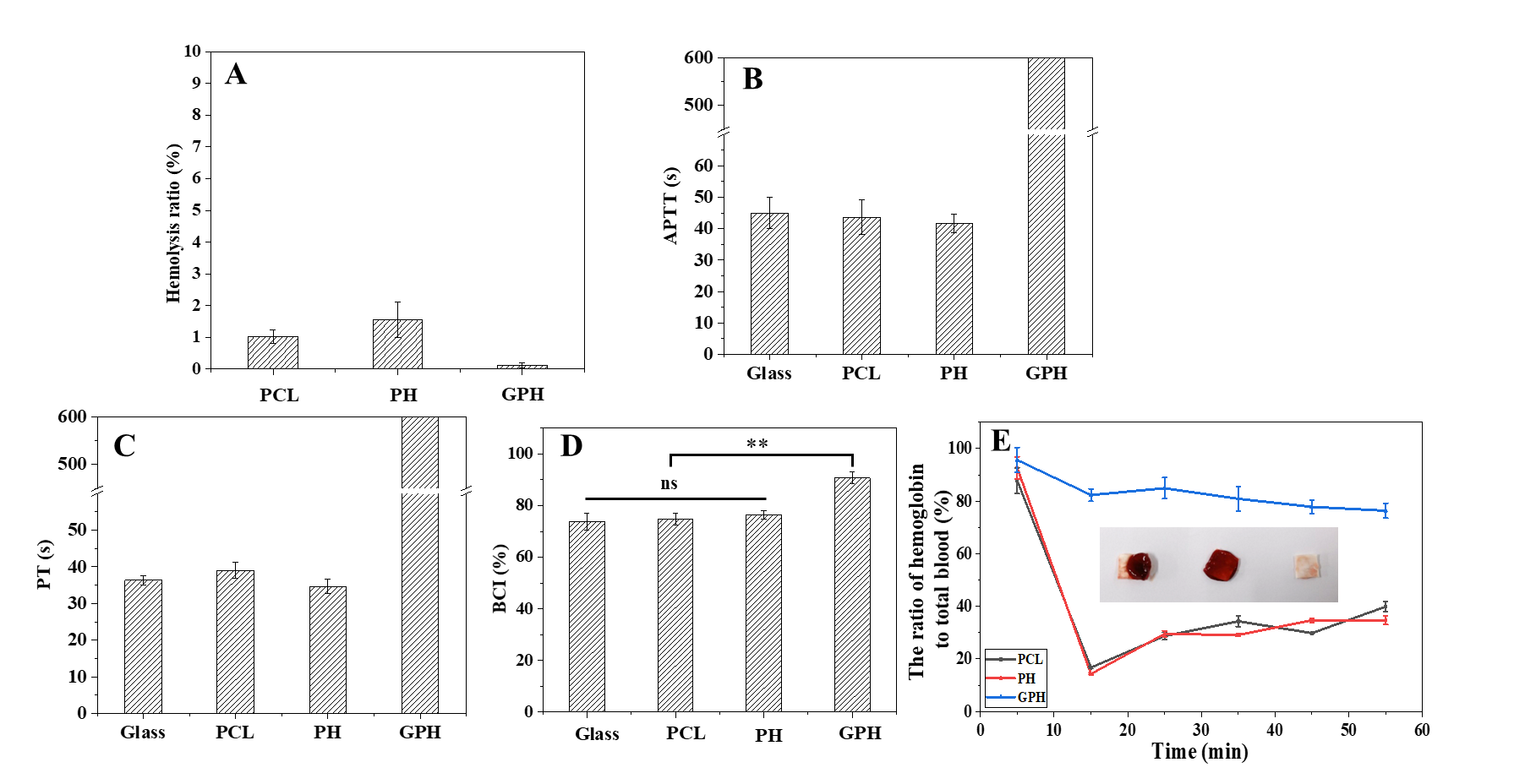


**Figure S5.** Blood compatibility and anticoagulant ability of GPH fiber scaffold. (A), (B),(C) and (D) represent respectively hemolysis rate, APTT, PT and BCI of different scaffolds. (E) Blood coagulation index change at different time after incubation with different scaffolds. The inset is image of scaffold (From left to right, represent PCL, PH and GPH) immersed in whole blood for 55 minutes. The data are shown as the means ± S.D. from three independent experiments. **p < 0.01 indicates significant differences between the indicated columns.
